# Proteomic Analysis of the Increased Stress Tolerance of *Saccharomyces cerevisiae* Encapsulated in Liquid Core Alginate-Chitosan Capsules

**DOI:** 10.1371/journal.pone.0049335

**Published:** 2012-11-09

**Authors:** Johan O. Westman, Mohammad J. Taherzadeh, Carl Johan Franzén

**Affiliations:** 1 School of Engineering, University of Borås, Borås, Sweden; 2 Industrial Biotechnology, Chalmers University of Technology, Göteborg, Sweden; Lawrence Berkeley National Laboratory, United States of America

## Abstract

*Saccharomyces cerevisiae* CBS8066 encapsulated in semi-permeable alginate or alginate-chitosan liquid core capsules have been shown to have an enhanced tolerance towards complex dilute-acid lignocellulose hydrolysates and the lignocellulose-derived inhibitor furfural, as well as towards high temperatures. The underlying molecular reasons for these effects have however not been elucidated. In this study we have investigated the response of the encapsulation on the proteome level in the yeast cells, in comparison with cells grown freely in suspension under otherwise similar conditions. The proteomic analysis was performed on whole cell protein extracts using nLC-MS/MS with TMT® labelling and 2-D DIGE. 842 and 52 proteins were identified using each method, respectively. The abundances of 213 proteins were significantly different between encapsulated and suspended cells, with good correlation between the fold change ratios obtained by the two methods for proteins identified in both. Encapsulation of the yeast caused an up-regulation of glucose-repressed proteins and of both general and starvation-specific stress responses, such as the trehalose biosynthesis pathway, and down-regulation of proteins linked to growth and protein synthesis. The encapsulation leads to a lack of nutrients for cells close to the core of the capsule due to mass transfer limitations. The triggering of the stress response may be beneficial for the cells in certain conditions, for example leading to the increased tolerance towards high temperatures and certain inhibitors.

## Introduction

In the search for a replacement for fossil fuels bioethanol comes out as one of the most promising alternatives. For sustainable production without interference with food production it is necessary to use lignocellulosic sources such as agricultural or forestry residues as raw materials [1]. However, the inherent recalcitrance of these materials makes extensive pretreatment and hydrolysis necessary for efficient release of fermentable sugars [2]. This often creates significant amounts of by-products that act as inhibitors of the subsequent fermentation, lowering the efficiency and feasibility of the process [3]–[5].

The most widely used microorganism for production of fuel ethanol, be it 1^st^ or 2^nd^ generation, is *Saccharomyces cerevisiae*. This yeast is capable of *in situ* detoxification of toxic hydrolysates, however, the inhibitor to cell ratio has to be low [6]. A low ratio can be achieved by increasing the local cell concentration, by cell immobilization or flocculation and cell recirculation [7], [8]. With a high local cell concentration, the ratio of inhibitors to cells becomes smaller locally and thus the cells can better handle the toxicity of a hydrolysate [9].

Cell immobilization can be done in a number of ways, but the one giving the highest local cell density is undoubtedly encapsulation in a semi permeable membrane. Local cell densities of several hundred grams dry weight per litre of capsule volume have been achieved [10]. Encapsulation of yeast cells has been shown to improve the fermentative performance in toxic lignocellulosic hydrolysates [8], as long as the inhibitors can be converted at a high rate [11]. Increased thermotolerance has also been observed upon encapsulation [12]. It is obvious that encapsulating cells will affect their growth and metabolism due to the close contact with other cells and due to the increased diffusion resistances that may lead to nutrient-limited conditions in the core of the capsule. It has been shown, that encapsulation leads to significantly lower cellular contents of RNA and protein as well as a lower RNA/protein ratio, and to higher cellular contents of trehalose, glycogen and total carbohydrates [13]. However, it is not clear how encapsulation affects the cells on a more molecular level, and how the responses facilitate increased tolerance to inhibitors or elevated temperatures. Furthermore, genome-wide investigations, on e.g. transcriptome or proteome level, of the physiological changes in yeast encapsulated in liquid core capsules have not yet been performed. A better understanding of the biochemical background of the improved tolerance may be used to design superior yeast strains, able to ferment toxic hydrolysates at high rates even without the need of an enclosing membrane.

Quantitative proteomics is of utmost importance for the understanding of changes in cellular physiology arising from different treatments of the cells, as different proteins, including post translationally modified variants, are directly linked to metabolic fluxes and cellular structure, and therefore ultimately determine the physiology. There are a number of different quantitative proteomic methods available, with two major types of protein separation, namely two dimensional gel electrophoresis (2-DE) and multidimensional liquid chromatography (multidimensional protein identification, MudPIT), often called nLC-MS/MS. For identification of proteins both methods depend on mass spectrometry in combination with database searches. For 2-DE one of the currently most popular methods is 2-D difference gel electrophoresis (2-D DIGE), where proteins from different samples are labelled with different fluorescent probes, enabling quantification of proteins from different samples in the same gel [14]. In MudPIT the most common in vitro labelling method is by isobaric mass tags, such as iTRAQ® or TMT®, that are applied after enzyme digestion of the protein samples to covalently label the peptides of different samples [15], [16]. The isobaric mass tags have different isotopic substitutions that, as the tags are cleaved off the peptides in the MS/MS mode, result in reporter ions of different weight, thus enabling quantification of proteins from different samples.

In this study, we compare the protein expression levels in yeast cells growing anaerobically either in liquid core capsules enclosed by alginate-chitosan membranes or in suspension, using both the 2-D DIGE approach and a MudPIT approach with TMT®. In addition to the overall elucidation of physiological changes in the cells due to encapsulation, the aim was to find possible reasons for the increased tolerance towards lignocellulosic derived inhibitors as well as for the enhanced yield of ethanol and lower glycerol yield of encapsulated yeast [13]. We show that encapsulation leads to general down-regulation of proteins involved in growth and protein synthesis, and up-regulation of proteins involved in both specific responses to nutrient limitation, as well as general stress responses. We conclude that the triggering of general stress responses is the underlying mechanism of the improved inhibitor tolerance of encapsulated yeast.

## Materials and Methods

### Yeast strain and medium

The *S. cerevisiae* CBS8066 obtained from Centraalbureau voor Schimmelcultures (Delft, the Netherlands) was used in all experiments. The strain was maintained on YPD agar plates with 10 g l^−1^ yeast extract (Scharlau), 20 g l^−1^ soy peptone (Fluka) and 20 g l^−1^ D-glucose (Fisher Scientific) as an additional carbon source.

Aerobic cultures for cell propagation were grown in 250 ml cotton-plugged conical flasks. Anaerobic batch cultivations were performed in 250 ml conical flasks equipped with a rubber stopper fitted with a loop trap filled with sterile water to permit produced CO_2_ to leave the flasks, and stainless steel capillaries for sample removal. The growth medium used for the batch cultivations was a defined glucose medium (DGM), as previously reported [17], with ergosterol (Sigma) added during anaerobic cultivations.

### Encapsulation procedure

The capsules were prepared by the liquid-droplet-forming method [18]. Yeast cells were grown in 100 ml DGM (50 g l^−1^ glucose) for 24 h in a shaker bath (130 rpm) at 30°C. Yeast from 50 ml of medium was harvested at 3500 g for 4 minutes and resuspended in 50 ml 1.3% (w/v) sterile CaCl_2_ (Scharlau) solution containing 1.3% (w/v) carboxymethylcellulose (CMC) (Aldrich) with average Mw of 250 kDa and degree of substitution 0.9. CMC increases the viscosity of the CaCl_2_ solution, thereby enhancing the formation of spherical capsules. A sterile solution containing 0.6% (w/v) sodium alginate (Product number 71238, Sigma) and 0.1% (v/v) Tween 20 (Sigma-Aldrich) was used for capsule formation. The surfactant Tween 20 improves the permeability of the capsule membrane, thereby preventing the capsules from rupturing due to CO_2_ formation during the cultivation [19].

Capsules were formed by dripping the CMC-yeast-CaCl_2_ solution into the stirred sodium alginate solution through syringe needles. The capsules were gelled for 10 minutes, washed with ultra-pure water for 10 minutes and hardened in 1.3% (w/v) CaCl_2_ solution for 20 minutes. The calcium-alginate capsules were thereafter treated in a 0.2% (w/v) low molecular weight chitosan (Product number 448869, Aldrich) solution with 300 mM CaCl_2_ in 0.040 M acetate buffer, pH 4.5, at a ratio of 1∶10 of capsules to solution for 24 hours. The treatment was performed in 0.5 l Erlenmeyer flasks in a water bath at 30°C at 130 rpm. Chitosan molecules are incorporated in the alginate matrix, thereby improving the capsules' strength by creating alginate-chitosan membranes [20].

### Cultivation and cell sampling

Approximately 15 ml of cell seeded capsules were cultivated aerobically in 100 ml DGM containing 40 g l^−1^ glucose for 24 hours in a shaker bath (130 rpm) at 30°C. The capsules were thereafter rinsed with sterile 0.9% NaCl (Scharlau) and transferred to fresh medium for another 7 hours. Fifty capsules were subsequently transferred to 100 ml of DGM, 40 g l^−1^ glucose, for anaerobic batch cultivations, giving a starting cell concentration of 0.77±0.04 g DW l^−1^ liquid volume. Samples for protein expression analysis were taken after 25.4±0.3 hours, when the glucose concentration had reached 12.6±0.7 g l^−1^. Cells from 8 capsules were washed out with ice-cold sterile ultra-pure water, and harvested at 10,000×g for 1 minute at 4°C. The pellet was immediately frozen in N_2_(l) and subsequently kept at −80°C until analysis.

Propagation of suspended yeast was started from aerobic 24 hours cultivations in 100 ml DGM (50 g l^−1^ glucose), by a 1% dilution into fresh DGM. After 23.5 hours, cells were harvested (3500 g, 4 minutes) and resuspended in 100 ml fresh DGM (40 g l^−1^ glucose) for anaerobic cultivations, at an initial cell concentration of 0.74±0.01 g DW l^−1^. The cells were grown anaerobically and samples for protein expression analysis were taken at 8.3±0.1 hours, when 15.8±0.1 g l^−1^ glucose remained in the cultivations. Cells were harvested by centrifugation of 20 ml cell suspension for 1 min at 3,500×g, 4°C, followed by washing of the cell pellet with ice-cold ultrapure water and centrifugation for 1 min at 10,000×g, 4°C. The pellet was immediately frozen in N_2_(l) and subsequently kept at −80°C until preparation of protein extracts and analysis.

After a series of trial cultivations, the sampling times were chosen to obtain samples when the residual extracellular glucose concentration and total amount of cells in the cultivations were approximately equal, and glucose was being consumed at constant rates. Free cells were sampled at a slightly higher residual glucose concentration, due to a higher biomass yield on glucose.

### Proteomic analysis by nLC-MS/MS and 2-D DIGE

Cells from five biological replicates each of free and encapsulated yeast were lysed and the proteins extracted and cleaned up prior to dividing the samples for 2-D DIGE and quantitative nLC-MS/MS.

For the nLC-MS/MS, precipitated protein pellets of 100 µg of each samples (three biological replicates each of free and encapsulated yeast) were digested with trypsin and labelled with a six-plex set of Tandem Mass Tag reagents following the manufacturer's instructions (Thermo Fisher Scientific). Nano LC-MS/MS analysis was performed on a LTQ Orbitrap Velos instrument (Thermo Fisher Scientific, Inc., Waltham, MA, USA) interfaced with an in-house constructed nano-LC column. MS data analysis was performed using Proteome Discoverer version 1.2 (Thermo Fisher Scientific).

2-D DIGE analyses [14] was performed across 5 gels. The samples were G-Dye labelled according to the manufacturers standard protocol (NH DyeAGNOSTICS), individual samples were labelled with G-Dye200 or G-Dye300 dyes using dye switching, while the internal standard was always G-Dye100 labelled. Isoelectric focusing was done in 24 cm pH 3–10 Nonlinear Imobiline DryStrips (GE Healthcare) on an Ettan IPGphore. The second dimension was run on an Ettan DALT II in in-house made polyacrylamide Bis-Tris gels. After 2D electrophoresis, the gels were scanned using the VersaDoc MP 4000 (BioRad) and the gel images were analysed using the Progenesis SameSpots software ver. 4.1 (Nonlinear Dynamics) for spot detection, spot quantification, inter-gel matching, and statistics. Spots with significant differences (Anova p<0.05) were selected for further identification, picked and digested with trypsin prior to identification using MALDI-TOF MS/MS.

Further details of the proteomic analyses are described in Methods S1. The nLC-MS/MS data associated with this manuscript may be downloaded from ProteomeCommons.org Tranche using the following hash: t7gxXL8oO/kgqNEGa2zWDwUVQKnEa90+l/W++pR6JKuwNXrY1m7hHCrqnVsNn6M0pv3w0aebnXgnl8vXv6N+YlyNFHcAAAAAAAACug =  = .

### Presentation of protein expression

The ratios between the abundances of proteins in suspended and encapsulated yeast are presented by one to three numbers in parentheses after the name of the protein. The first, and in most cases the only, number represents the ratio (fold change, FC) obtained by n-LC-MS/MS. When applicable, this is followed by the average ratio obtained from 2-D DIGE spots with unique significant protein hits, and lastly the average ratio obtained from spots with significant hits for more than one co-migrating proteins in 2-D DIGE. Thus, Tps1p (1.77, 1.59, 1.63) indicates a FC of 1.77 estimated by the nLC-MS/MS approach, an average FC of 1.59 estimated from spots with a unique significant hit for Tps1p, and an average FC of 1.63 estimated from spots with significant hits from both Tps1 and co-migrating proteins.

### Analytical methods

The amounts of metabolites were quantified by HPLC using an Aminex HPX-87H column (Bio-Rad) at 60°C with 5 mM H_2_SO_4_ as eluent at a flow rate of 0.6 ml min^−1^. A refractive index detector was used for the detection and quantification of glucose, acetic acid, lactic acid, glycerol and ethanol.

The cell dry weight was measured in predried and preweighed glass tubes. Cells were separated by centrifugation and washed once with ultra-pure water before drying for approximately 24 h at 105°C. Cells from capsules were released by crushing the capsule followed by extensive washing of the capsule debris with ultra-pure water.

### Statistics, yields and elemental balance calculations

The biomass and metabolite yields as well as the carbon balance were calculated from the determined concentrations at the end of the fermentations, i.e. the time of sampling for proteome analysis. Error intervals are given as 95% confidence intervals of the mean, unless otherwise stated.

## Results and Discussion

### Fermentation performance of free and encapsulated cells

**Figure 1:**
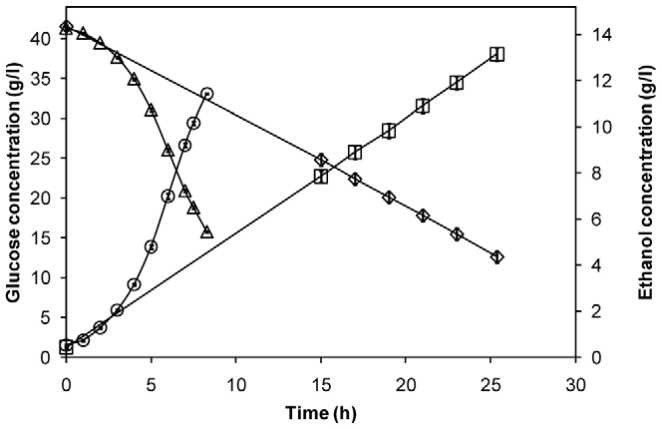
Fermentation profiles of encapsulated and free *S. cerevisiae*. Glucose and ethanol concentration profiles of encapsulated (◊, □) and free (▵, ○) cells during anaerobic batch cultivations.

Free and encapsulated cells were grown anaerobically in shake flasks starting at the same initial cell concentration, 0.75±0.02 g DW l^−1^ medium volume. The cells were sampled at residual glucose concentrations of 14.2±1.2 g l^−1^ while consuming glucose and producing ethanol at constant rates (Figure 1). The encapsulated cells consumed glucose markedly slower than the free cells, with a linear glucose consumption profile never reaching a true exponential growth phase. The sampling times were chosen as a compromise between residual extracellular glucose concentration and total cell amount in the cultivations in order to minimize the differences between the two modes of cultivation. The two systems are distinctly different, and the resulting differences in the proteome and other variables must be viewed as integrated responses to both direct and indirect effects of the encapsulation.

The yields of major metabolites and biomass were significantly different between cells grown in the two ways. The encapsulated cells had higher ethanol yield than the free cells, whereas free cells had higher glycerol, acetate and biomass yields (Table 1), similar to what has been observed in previous studies of encapsulated yeast [9]. Interestingly, low levels of lactate, the end-product of methylglyoxal degradation in anaerobic cultures, were also detected towards the end of the cultivations with encapsulated yeast (Table 1). Similar observations have been made in retentostat cultures with yeast growing extremely slowly [21], [22].

**Table 1:** Key yields during anaerobic batch cultivations of free and encapsulated *S. cerevisiae*.

	Y_SE_	Y_SGly_	Y_SAce_	Y_SLac_	Y_SBiomass_	Carbon recovery (%)
Free	427±4	56±1	5±0	n.d.	74±2	98.8±1.0
Encapsulated	439±3	48±2	2±0	3±0	41±3	96.3±0.8

Yields are shown as mg product per g consumed glucose from the start until the sampling of cells in the anaerobic batch cultivations. The molar CO_2_ production was assumed to be the same as the sum of ethanol and acetate. Error intervals shown are 95% confidence intervals, with n = 5. Y_SE_ – Ethanol yield, Y_SAce_ – Acetate yield, Y_SGly_ – Glycerol yield, Y_SLac_ – Lactate yield, Y_SBiomass_ – Biomass yield, n.d. – not detected.

**Figure 2:**
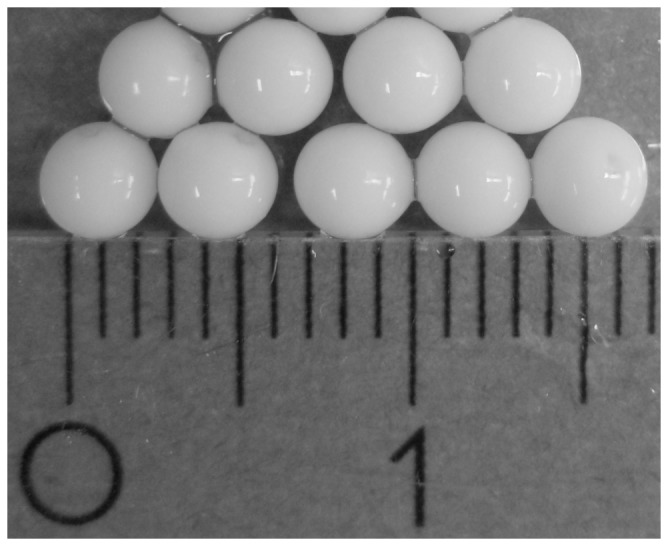
*S. cerevisiae* encapsulated in alginate chitosan capsules. Capsules full of cells at the time of sampling for proteome analysis. Major unit of the ruler is in centimetres.

The slower growth rate and lower biomass yields of encapsulated cells are likely an effect of mass transfer limitations to the cells in the middle of the capsules. The amount of biomass per capsule was 1.5±0.1 mg at the start of the cultivation, which increased to 3.9±0.1 mg per capsule at the time of sampling. This corresponds to approximately 300 g DW (l capsule volume)^−1^. It has previously been shown, that for a flocculating yeast strain mass transfer limitations occur in flocs larger than 100 µm [23] and that mass transfer into capsules decrease as the capsules fill up with cells [13]. After chitosan treatment, the capsules used in this study had a mean diameter of 3.63±0.03 mm. At the time of sampling, the capsules were full of cells that were likely not moving around freely (Figure 2). This undoubtedly gives rise to mass transfer limitations into the interior of the capsule, considering the fact that glucose diffusivity in water alone is 600 µm^2^ sec^−1^ [24]. Consumption of glucose by cells close to the membrane also leads to decreasing concentrations closer to the core of the capsule. For these reasons there are likely also physiological differences between cells along the radius towards the centre of the capsule. However, for the proteomic analysis in this study we consider only the mean values of all protein levels in the cell population, since cells were quantitatively washed out of the capsules at the time of sampling. The possible error introduced by this methodology would be an underestimate of the differences between cells in the core of the capsule and the suspended cells.

### Protein expression in response to encapsulation

**Figure 3:**
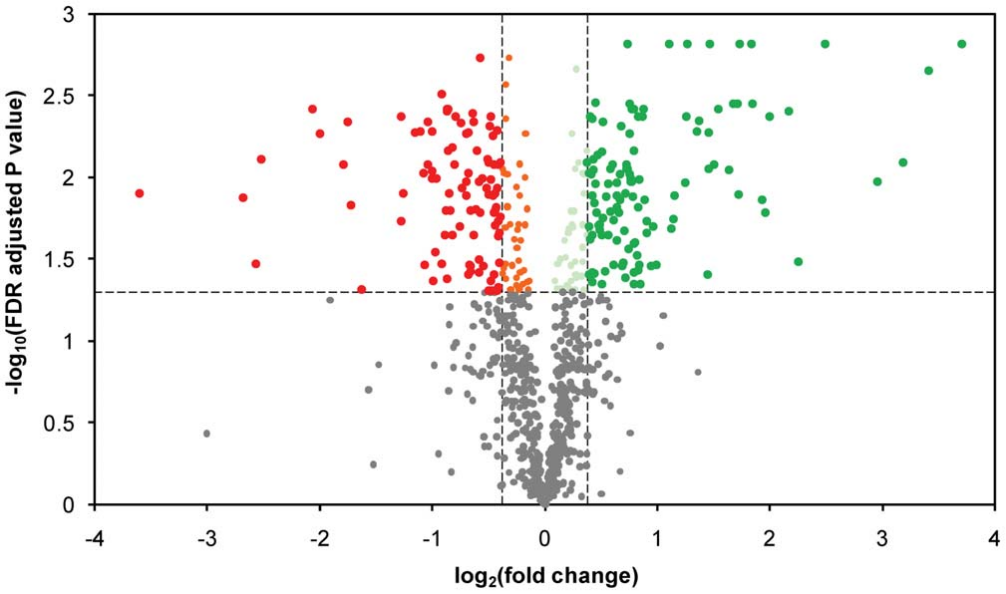
Proteome based pair-wise comparison of encapsulated and free *S. cerevisiae*. Volcano plot illustrating the distribution of all proteins identified with the nLC-MS/MS approach. Significantly up- and down-regulated proteins (|fold change| ≥1.3, x-axis; FDR adjusted p value≤0.05, y-axis) are highlighted in green and red respectively. Statistically up- and down-regulated proteins with non-significant biological changes (|fold change| <1.3) are shown in light green and orange, respectively, and proteins with non-significant differences between the free and encapsulated yeast are shown in grey.

**Figure 4:**
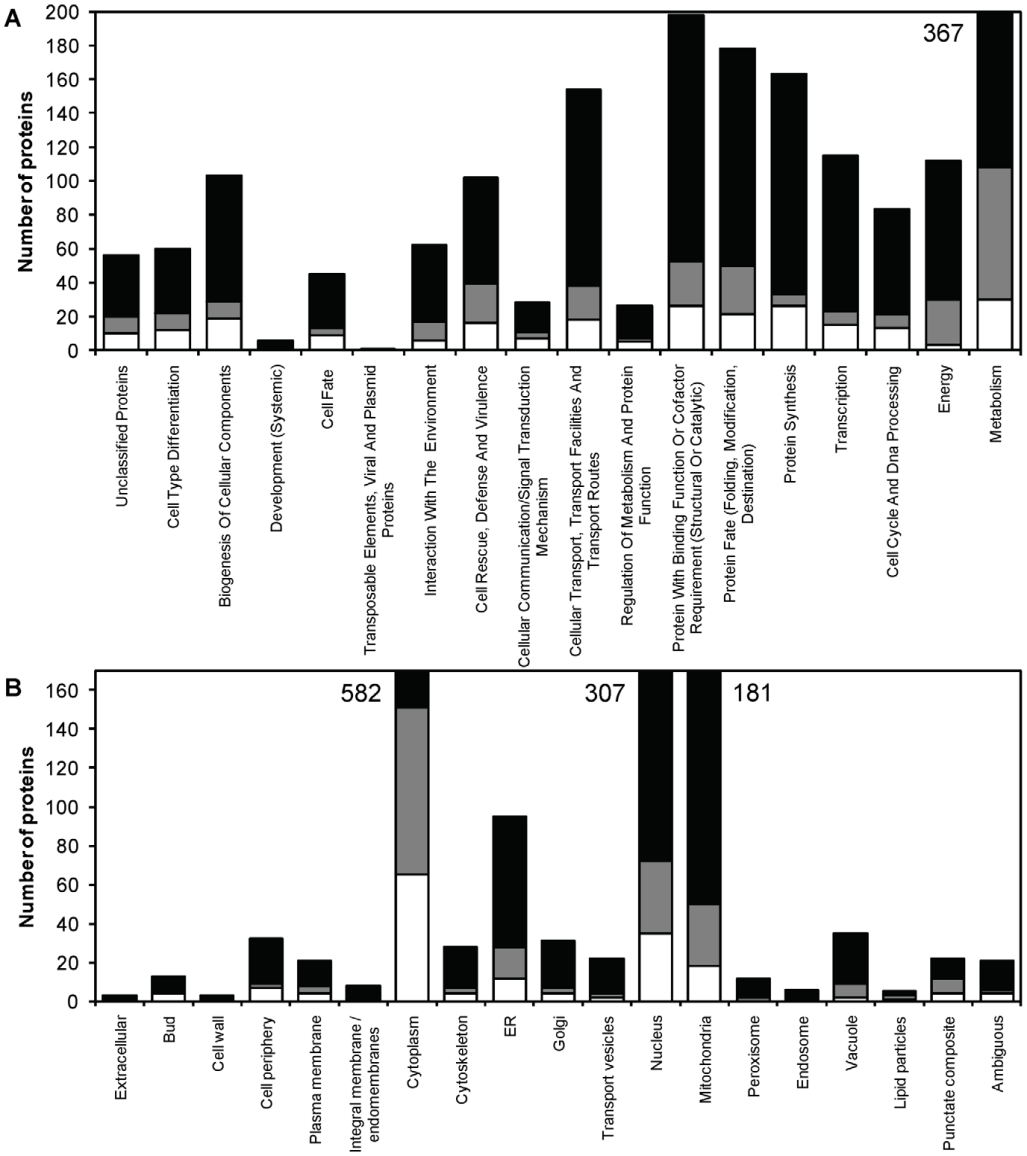
Functional classification and cellular localization of proteins identified by the nLC-MS/MS approach. Distribution of functional categories (A) and cellular localizations (B) of identified proteins in encapsulated and free *S. cerevisiae*, showing the number of proteins in the respective fold change class (non-regulated – black, up-regulated – grey, down-regulated – white) per functional category and cellular localization, respectively. Numbers next to bars indicate the total number of proteins in the category when extending past the y-axis range.

Of the 842 proteins identified with nLC-MS/MS (Table S1), 116 were up-regulated and 95 down-regulated according to the set significance criteria (Figures 3 and 4). The large number of differentially expressed proteins indicates that the environmental conditions are quite different for yeasts growing in the capsules and in suspension. The localization and functional category of differentially expressed proteins were determined using the FunCatDB software (Figure 4, Table S2). Notably, down-regulated proteins were over-represented when compared to up-regulated in cellular locations important for cell growth and division, such as the cell periphery, bud, cytoskeleton and Golgi. This was also the case for the functional categories transcription and protein synthesis (Figure 4, Table 2). These results mirror the decreased growth inside the capsules, visualized by the decreased biomass yield (Table 1) and slower glucose consumption rate (Figure 1). Up-regulated proteins were instead over-represented mainly in the cytoplasm and mitochondria (Figure 4B), where many proteins involved in the two categories energy and metabolism are localized (Figure 4A, Table 3, Figure S3). In these functional categories, up-regulated proteins were also over-represented.

**Table 2:** Functional categories enriched among down-regulated proteins.

Category	Sub-category	p value	Proteins
Metabolism (30)	Metabolism of the cysteine – aromatic group (5)	7.7E-03	Aro2p Aro7p Gly1p Cys4p Gcv1p
	Metabolism of glycine (2)	6.3E-03	Gcv1p Gly1p
Protein Synthesis (26)	Ribosome biogenesis (15)	7.2E-05	Tif5p Ygr054wp Drs1p Rna1p Ria1p Nsr1p Rpl16bp Rpl17ap Rps24ap Rps21ap Rpl31bp Nog1p Rpl19ap Prp20p Ubi3p
	Ribosomal proteins (10)	4.5E-03	Ubi3p Rps21ap Rpl17ap Rpl19ap Rpl31bp Drs1p Rps24ap Ygr054wp Nsr1p Rpl16bp
	Translation (11)	8.2E-08	Egd1p Eft1p Cdc33p Ria1p Efb1p Hyp2p Pab1p Ygr054Wp Tif5p Tif3p Caf20p
	Translation initiation (5)	3.4E-04	Hyp2p Ygr054Wp Tif3p Cdc33p Tif5p
	Translation elongation (3)	3.9E-03	Ria1p Efb1p Eft1p
Protein fate (21)	Protein folding & stabilization (6)	3.0E-03	Cct5p Cct2p Ydj1p Zuo1p Sti1p Caj1p
Protein w. binding function (26)	Protein binding proteins (13)	9.1E-03	Srv2p Scs2p Cct2p Hyp2p Zuo1p Sti1p Egd1p Pea2p Rvs167p Bbc1p Ssz1p Cct5p Abp1p
	RNA binding proteins (9)	2.6E-03	Pab1p Gbp2p Rpl16Bp Nop13p Scp160p Nsr1p Bfr1p Tma22p Arc1p
Cellular transport (19)	RNA transport (6)	2.1E-03	Rna1p Scp160p Prp20p Gbp2p Pab1p Arc1p
Cellular communication (7)	Small GTPase mediated signal transduction (5)	1.9E-03	Srv2p Ras2p Zeo1p Cla4p Pea2p
Cell rescue, defence and virulence (16)	Stress response (16)	1.3E-03	Cct5p Nsr1p Zuo1p Zeo1p Gbp2p Rhr2p Ssz1p Cct2p Ras2p Sod1p Rvs167p Yhb1p Stm1p Sti1p Egd1p Ydj1p
	Unfolded protein response (6)	6.5E-04	Sti1p Ssz1p Cct5p Egd1p Zuo1p Cct2p

Enriched (p<0.01) functional categories among down-regulated proteins in encapsulated yeast, as analysed using the MIPS functional category enrichment tool (FUNCAT, http://www.helmholtz-muenchen.de/en/mips/projects/funcat). The number of proteins in each category is shown in parentheses.

**Table 3:** Functional categories enriched among up-regulated proteins.

Category	Sub-category	p value	Proteins
Metabolism (32)	Metabolism of glutamine (2)	4.9E-03	Gln1p Fas1p
	Phosphate metabolism (21)	2.2E-05	Rix7p Tpk1p Ugp1p Hsp78p Cka1p Vph1p Ypk1p Pex6p Ssa1p Pro1p Tpk2p Rli1p Hxk1p Ssb2p Tps2p Glc7p Glk1p Stt4p Rpt5p His2p Hsp104p
	C-compound and carbohydrate metabolism (35)	2.1E-12	Glk1p Emi2p Adh5p Pmt7p Hsp12p Ach1p Ayr1p Gsy2p Ybr056Wp Glc3p Tps1p Adh1p Gph1p Gre3p Gln1p Tdh1p Mal62p Tsl1p Dld2p Gnd1p Tps2p Hxk1p Kgd1p Ald4p Ugp1p Dpm1p Nth1p Uga1p Ynr071Cp Gdb1p Dsf1p Gsy1p Pgm2p Glc7p Tal1p
	Sugar, glucoside, polyol and carboxylate metabolism (10)	2.1E-06	Tps1p Tdh1p Pgm2p Kgd1p Nth1p Tsl1p Mal62p Gre3p Tal1p Ugp1p
	Sugar, glucoside, polyol and carboxylate anabolism (7)	2.8E-06	Nth1p Ugp1p Tsl1p Tps1p Mal62p Tal1p Pgm2p
	Sugar, glucoside, polyol and carboxylate catabolism (9)	1.3E-05	Nth1p Mal62p Kgd1p Tal1p Tps1p Tdh1p Pgm2p Gre3p Ugp1p
	Polysaccharide metabolism (7)	1.8E-04	Glc3p Gln1p Gdb1p Gsy1p Gph1p Dpm1p Gsy2p
	Glycogen metabolism (2)	3.3E-03	Gsy1p Gsy2p
	Glycogen anabolism (2)	3.3E-03	Gsy1p Gsy2p
	Lipid, fatty acid and isoprenoid metabolism (22)	8.7E-09	Stt4p Erg13p Dpm1p Erg11p Erg3p Cat2p Pdx3p Scs3p Fas1p Slc1p Fas2p Mcr1p Erg25p Ayr1p Ypk1p Ach1p Hsp12p Yml131Wp Ole1p Ura8p Fas3p Mrs6p
	Fatty acid metabolism (4)	9.0E-04	Fas1p Fas2p Fas3p Ole1p
	Tetracyclic and pentacyclic triterpenes metabolism (5)	4.8E-04	Erg25p Erg3p Erg11p Erg13p Mcr1p
Energy (27)	Pentose phosphate pathway (4)	9.0E-04	Gnd1p Tal1p Ynr034Wp Pgm2p
	Alcohol fermentation (3)	1.6E-03	Adh1p Adh5p Ald4p
	Metabolism of energy reserves (13)	1.5E-11	Nth1p Tps2p Gsy1p Gph1p Glc7p Gdb1p Gsy2p Mal62p Ugp1p Tsl1p Glc3p Tps1p Pgm2p
Protein w. binding function (31)	Nucleotide/nucleoside/nucleobase binding (12)	9.0E-04	Ssa1p Rix7p Tpk2p Hnt1p Tpk1p Hsp104p Pex6p Hsp78p Rpt5p Rli1p Ssb2p Lap3p
	Cyclic nucleotide binding (2)	2.0E-03	Tpk1p Tpk2p
	FAD/FMN binding	8.9E-03	Mcr1p Oye3p
Cellular transport (24)	Electron transport (7)	8.3E-04	Oye3p Mcr1p Vma2p Vma13p Atp7p Vma6p Vph1p
	Transport ATPases (5)	3.1E-03	Vma6p Vma2p Vph1p Atp7p Vma13p
Cell rescue, defence and virulence (24)	Stress response (20)	2.0E-04	Sip18p Tps1p Ssb2p Tps2p Mcr1p Hsp26p Mdj1p Hsp78p Pst2p Tsl1p Nth1p Hsp12p Hsp104p Pep4p Def1p Glc7p Ssa1p Aip1p Cka1p Gre3p
	Heat shock response (3)	5.7E-03	Glc7p Hsp12p Gre3p
	Unfolded protein response (5)	8.9E-03	Ssa1p Mdj1p Hsp78p Hsp26p Ssb2p
Interaction with the environment (11)	Homeostasis of protons (5)	1.8E-03	Vma2p Vma13p Vma6p Atp7p Vph1p

Enriched (p<0.01) functional categories among up-regulated proteins in encapsulated yeast, as analysed using the MIPS functional category enrichment tool (FUNCAT, http://www.helmholtz-muenchen.de/en/mips/projects/funcat). The number of proteins in each category is shown in parentheses.

### Nutrient limitation in capsules

**Figure 5:**
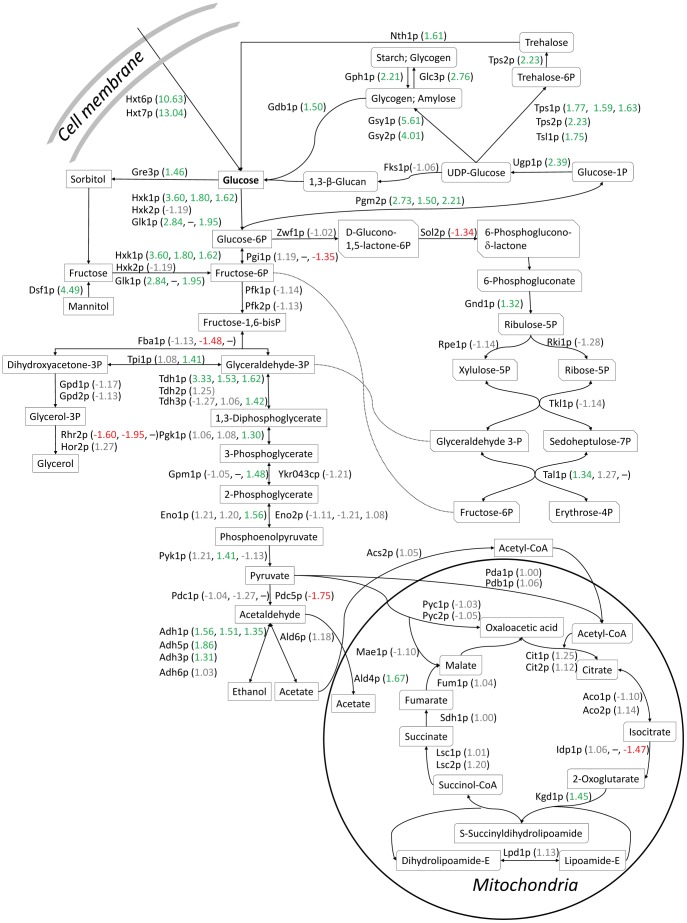
The proteomic response on the central carbon metabolism upon encapsulation of yeast. The central carbon metabolism is presented with up-regulated proteins with fold changes (encapsulated cells compared to free cells) in green, down-regulated proteins with fold changes in red and unaffected proteins with the measured fold changes in grey. The first number represents the fold change obtained by n-LC-MS/MS. Where applicable, this is followed by the average fold change obtained from 2-D DIGE spots with unique significant protein hits, and the average fold change obtained from spots with significant hits for co-migrating proteins in 2-D DIGE.

Of utmost importance for the production of bioethanol are the metabolism and energy turnover of the yeast. The differences in metabolite yields between the two modes of cultivation indicate that the metabolism of the yeast changed significantly upon encapsulation (Table 1). Of the 112 quantified proteins classified as being involved in the functional category “energy”, a total of 27 were up-regulated and only 3 were down-regulated. Included in this category are e.g. parts of the central carbon metabolism such as glycolysis and gluconeogenesis, the pentose phosphate pathway, the TCA cycle and the trehalose and glycogen metabolism (Figure 5), all of which are localized to the cytoplasm and/or mitochondria.

The two most up-regulated proteins in encapsulated cells were the high affinity hexose transporters Hxt6p (10.63) and Hxt7p (13.04). The expression of these transporters is repressed by high glucose levels and they have high expression levels on non-fermentable carbon sources and at low concentrations of glucose [25], [26]. Several enzymes in the glycolytic pathway were significantly up-regulated, in many cases seen also using the 2-D DIGE method. Only one, Pdc5p, was down-regulated (Figure 5). The strong up-regulation of Hxk1p and Glk1p in the encapsulated yeast is notable, since their expressions, like the mentioned hexose transporters, are known to be repressed by glucose [27]. These are only some examples of the up-regulation of glucose-repressed proteins in the encapsulated cells and together they provide strong evidence for carbon limitation inside the capsules.

Another enzyme in the glycolytic pathway, Tdh1p catalysing the oxidative phosphorylation of glyceraldehyde-3-phosphate to 1,3-bisphosphoglycerate, is known to be expressed during stationary phase and other conditions of slow growth, while the Tdh2p and Tdh3p are detected in the exponential phase [28]. Tdh1p was detected at significantly higher levels in the encapsulated yeast than in the free cells, while there was no significant difference for Tdh2p and Tdh3p (Figure 5).

The cytoplasmic alcohol dehydrogenases Adh1p and Adh5p, and the glucose-repressed mitochondrial Adh3p, that all reduce acetaldehyde to ethanol, were up-regulated in the encapsulated cells (Figure 5). This is likely an effect of a higher maintenance energy requirement due to the low growth rate [29] and the increased anaerobicity. The glycerol-3-phosphatase Rhr2p was down-regulated, in line with the observed lower yield of glycerol for the encapsulated cells, which is to be expected since glycerol production is generally growth related when cells are not osmotically stressed.

The proteins involved in synthesis and utilization of the storage carbohydrates trehalose and glycogen were up-regulated (Figure 5). This is in accordance with previous reports of increasing trehalose and glycogen levels in encapsulated yeast as the capsules filled up with cells (4 and 4.5-fold increase, respectively, in the end compared to the beginning of a series of 20 sequential batches) [13]. Trehalose and glycogen are important storage carbohydrates that accumulate in slowly growing or starved yeast, but trehalose is also an important protector of membranes and proteins against various stresses such as cold, heat, and starvation [30], [31].

In addition to carbon starvation, it is possible that the cells in the middle of the capsules also experienced nitrogen and phosphate limitation, since both glutamine and phosphate metabolism were significantly up-regulated in the encapsulated cells (Table 3). Other indications of nutrient limitation are the up-regulation of heme-repressed proteins, such as Hem13p (2.91, 1.51), Ole1p (3.88), Anb1p (2.74) and Erg11p (1.34) [32], and down-regulation of proteins repressed by anaerobic conditions, such as Sod1p (−1.68) [33]. This shows that oxygen was absent in the capsules, meaning that the environment inside the capsule was more strictly anaerobic than in the suspension shake flasks. This could be a contributing factor to the observed increase in ethanol yield and decrease in biomass yield. Despite using air locks and rubber stoppers, some oxygen may diffuse into the shake flask culture.

### Down-regulated protein synthesis in encapsulated yeast

Another variable likely to change with a change in nutrient availability is the growth, and hence, also the protein synthesis should be affected. Previous reports have stated that both the total protein levels and the total RNA levels (with ribosomal RNA as the main contributor) decrease in prolonged growth of encapsulated yeast [13]. Of the identified proteins 163 were related to protein synthesis and 103 to ribosome biogenesis according to the FunCat analysis. Of these proteins, 26 and 15 were down-regulated, respectively (Table 2, Figure 4A), for example the most down-regulated protein Drs1p (−12.11), involved in ribosome assembly and function.

In the PRIMA analysis of the genes coding for regulated proteins, the usage of transcription factor Abf1p was enriched among the down-regulated proteins (Figure S4). Among other things, Abf1p is involved in initiation of DNA replication as well as activation of transcription of many genes coding for ribosomal proteins [34]. In cells immobilized in alginate gel beads, the levels of ribosomal proteins have instead been seen to be up-regulated [35], showing that there are differences between the physiological responses to immobilization in porous gels and to liquid core encapsulation.

Certain proteins in the unfolded protein response (UPR) related to growth were also down-regulated, e.g. Ssz1p (−1.49) and Zuo1p (−1.34) that act together in a complex involved in ribosome biogenesis [36]. Two other down-regulated UPR proteins belong to the cytosolic chaperonin Cct ring complex, Cct2p (−4.19) and Cct5p (−1.82). This complex is required for assembly of actin and tubulins [37]. The slower growth of the encapsulated yeast would decrease the need of new actin assembly and of the Cct complex, hence its decreased expression. Other proteins involved in new actin assembly were also down-regulated, such as Bbc1p (−1.57), involved in the reorganization of the actin cytoskeleton [38], and Tpm1p (−1.80), involved in the assembly of actin cables [39], while actin itself, Act1p (1.13), showed invariant expression.

### Stress response proteins in encapsulated yeast

Considering the seemingly starvation-stressed cells, the expression of stress response proteins are of particular interest to study. Of the 91 proteins in the data set identified as stress response proteins according to the FunCat analysis, 21 were up-regulated, while 16 were down-regulated. Notable is that proteins under the control of the transcription factors Msn2 and Msn4 were enriched in the encapsulated yeast (Figure S4). These two transcription factors are activated in stress conditions and are an important part of the so called Environmental Stress Response (ESR) [40]. In accordance with the previously reported increased heat tolerance of similarly encapsulated cells [12], three proteins (Glc7p (1.58), Hsp12p (1.99) and Gre3p (1.46)) out of the five identified as involved in the “heat shock response” were up-regulated (Table 3), while the other two showed invariant (Get3p (1.17)) or statistically insignificantly increased (Bcy1 (1.39)) expression. This, together with the increase in intracellular trehalose, give the encapsulated cells an advantage compared to free cells in the response to an increased temperature. Among the proteins classified as involved in the response to osmotic and salt stress both up-regulated (Sip18p (7.77), Hsp12p (1.99) and Aip1p (1.78)) and down-regulated (Rhr2p (−1.60, −1.95)) proteins were found. This would indicate that the cells suffered from osmotic stress inside the capsules, were it not for the down-regulation of glycerol production. A more plausible explanation for the apparent osmotic response is cross talk between nutrient starvation and other environmental stress responses.

### Proteins involved in the response to furfural

One of the major benefits of using encapsulated yeast in 2^nd^ generation bioethanol production is its ability to tolerate otherwise too toxic dilute-acid hydrolysates. The tolerance of encapsulated yeast towards high levels of the pentose-derived furan aldehyde inhibitor furfural has been specifically studied [9]. Many of the proteomic responses to encapsulation observed in this study were similar to those in proteomic studies made on cells exposed to furfural [41], [42]. The improved tolerance of the encapsulated cells can therefore plausibly be ascribed, at least partly, to the cells being in a state more prepared for the stress caused by the inhibitor. The reduction of furfural to furfuryl alcohol is an important part of *S. cerevisiae*'s detoxification of lignocellulosic hydrolysates and this is believed to be performed mainly by the alcohol dehydrogenases [41], [43]. It has been shown that the levels of Adh1p and Adh5p were increased in the presence of furfural [41], why an already increased level, as found in this study, would make the introduction of furfural seem less harsh to the cells and thus require less severe adaptations.

Lin et al. [41], [42] suggested that yeast cells require an increased chaperone capacity when exposed to furfural, due to an increased amount of partially unfolded proteins. Of the up-regulated UPR proteins in our study, four (Ssa1p (1.32), Ssb1p (1.77), Hsp78p (1.39) and Ssb2p (1.71, –, 1.42)) belonged to the Hsp70 family, which has been shown to be up-regulated in the response to furfural [41]. In addition to these, the co-chaperone Mdj1p (1.31) and small heat shock protein Hsp26p (3.11) were also up-regulated. Down-regulated UPR proteins were instead mainly related to the growth of the cells (see above). Taken together it is probable that the up-regulation of chaperones does indeed help encapsulated cells to cope with the stress caused by furfural.

### 2-D DIGE support nLC-MS/MS results

**Figure 6:**
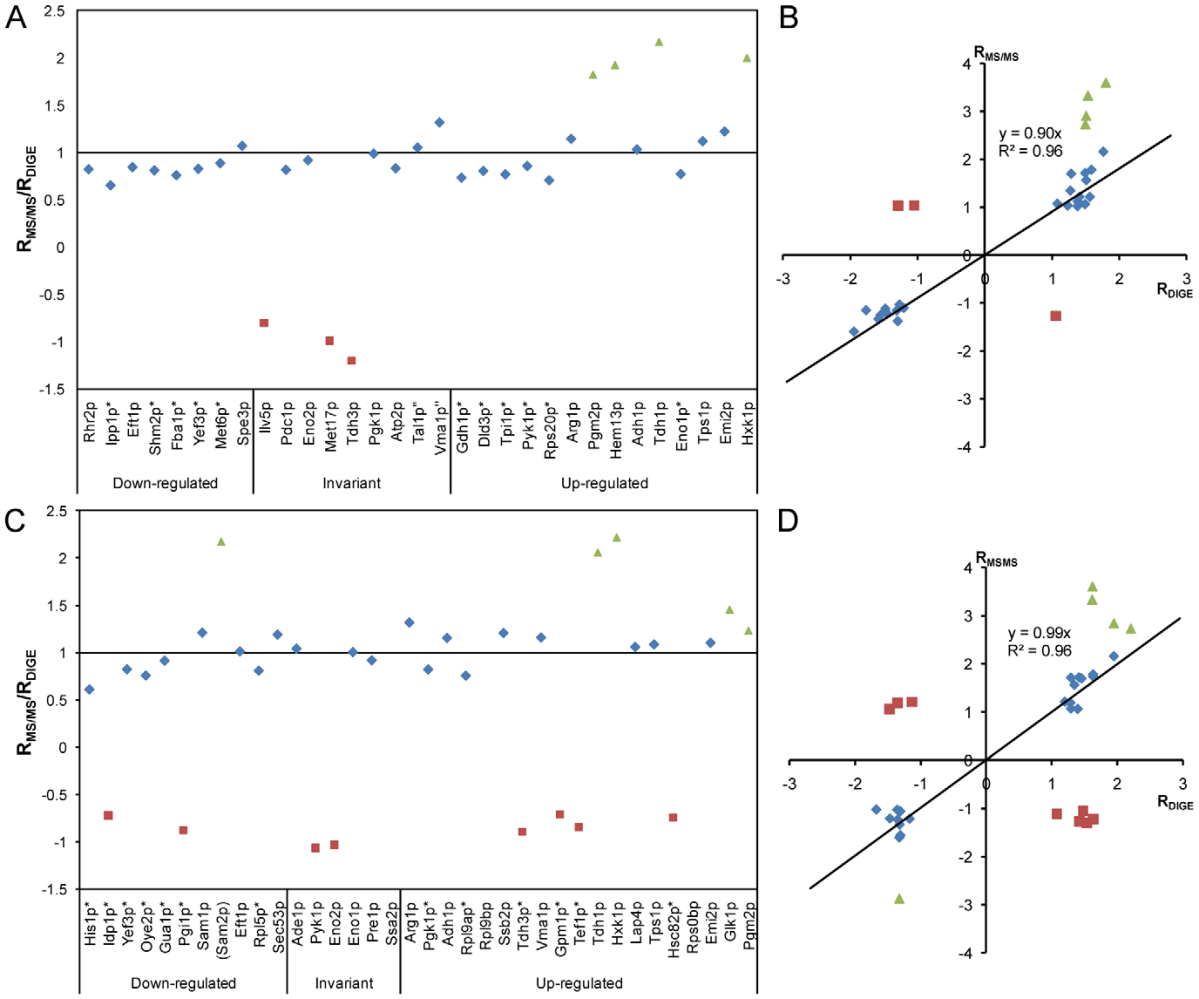
Correlation between the proteomic methods 2-D DIGE and nLC-MS/MS with TMT®. Correlation between the average ratios of 31 proteins (with single significant hits in spots on the gel) (A and B) and 33 co-migrating proteins (with two or more significant hits per spot on the gel, giving uncertainties in the quantification of each individual protein in the spot) (C and D) proteins obtained by 2-D DIGE (R_DIGE_) and nLC-MS/MS (R_MS/MS_). A and C, the ratios obtained by nLC-MS/MS divided by the mean ratios obtained by 2-D DIGE, for single significant hit spots and spots with co-migrating proteins respectively. Triangles indicate extremely up-regulated proteins (fold change >2.5) as measured by the nLC-MS/MS approach and squares indicate proteins showing different sign of the fold change in the two approaches. The proteins were sorted by increasing fold change values obtained by the 2-D DIGE approach and divided into three groups, depending on the expression according to 2-D DIGE. Proteins marked with * had invariant expression in the nLC-MS/MS approach, and those marked with “were up-regulated. Proteins in parentheses (Sam2p) had extremely large RSD among the replicates in nLC-MS/MS and missing values indicate that the protein was not detected in the nLC-MS/MS approach (Ssa2p, Rpl9bp, Rps0bp). B and D, correlation plots of the ratios obtained by DIGE (x-axis) against the ratios obtained by nLC-MS/MS (y-axis), for unique (B) and co-migrating (D) protein spots, respectively. Extremely up- or down-regulated proteins (triangles) as well as the three and eight proteins showing different expression with the two methods (squares) were excluded from the calculation of the correlation.

In addition to the nLC-MS/MS with TMT® labelling, relative quantification of proteins from cells encapsulated in alginate-chitosan gel membranes compared to free cells was carried out also with 2-D DIGE. In this approach 103 spots with differential protein expression (Anova p<0.05) were chosen for subsequent MALDI TOF/TOF analysis for protein identification. Proteins were identified in 100 spots, of which 93 had highly significant hits, with a total of 52 different proteins detected (Table S3). After sorting out spots containing more than one identified protein, 59 spots with a total of 31 different proteins remained. Due to e.g. protein processing and posttranslational modifications, the same protein can be present in a number of spots [44]. For example, the abundant glycolytic proteins Hxk1p, Pdc1p and Pyk1p were found as the only protein in five spots each. The average FC values of these 31 proteins from the 59 spots were compared with the values from the nLC-MS/MS approach by dividing the ratios obtained for the same proteins by 2-D DIGE with that obtained by nLC-MS/MS (Figure 6A and B). The comparison between 24 of the 31 proteins centred close to 1 (0.91±0.07), the value expected for optimal correlation. The proteins with the highest FC values with the nLC-MS/MS approach (FC>2.5, triangles in Figure 6) were detected at lower FC in the 2-D DIGE approach. Three more outliers were found that showed different sign of the FC in the two approaches (squares in Figure 6). However, none of these showed significant changes in either of the two approaches, meaning that they centred close to −1. The average ratio determined by nLC-MS/MS followed a linear relationship with the average ratio obtained by 2-D DIGE, R_MS/MS_ = 0.90 R_DIGE_, with an r^2^ value of 0.96 (Figure 6B).

In 34 of the 93 spots with significant protein hits, more than one protein was identified with significance. Such co-migration [44] is known to affect the accuracy of the quantification [45]. A total of 36 different proteins were found of which 15 were found also in the spots with only one significant hit, and 3 which were not identified in the nLC-MS/MS approach. By assigning the same FC to all significant protein hits in each spot and calculating the average ratio of the same protein from all spots with co-migrating proteins, a good correlation with the corresponding proteins quantified with the nLC-MS/MS method was obtained (Figure 6C and D). After sorting the proteins as described in the previous section, 20 of the 33 proteins had a good correlation between the two proteomic methods, with an average ratio, R_MSMS_/R_DIGE_, of 1.00±0.09 (Figure 6C). A linear relationship R_MS/MS_ = 0.99 R_DIGE_, with an r^2^ value of 0.96, was obtained (Figure 6D). It should be noted that there were 8 proteins showing different sign of the FC for the proteins quantified in spots with more than one significant protein hit, and of these, 5 proteins showed significant changes in one of the methods. Therefore it is important to keep track of whether the protein ratio is deduced from a spot with one or more significant protein hits when analysing data from 2-D DIGE.

On average, the two different methods gave similar expression values and the combination of the methods is thus a good way of verifying proteomic results. A comparison of DIGE quantification with metabolic labelling quantification of spots picked from a gel showed correlations similar to what was observed in our study [46].

A FunCat localization analysis of the proteins detected as single proteins in the spots on the gels showed that all of them localized to the cytoplasm, nucleus, mitochondria or vacuole. This showed a drawback of the 2-D DIGE approach when it comes to proteome-wide studies. Mainly abundant proteins are detected, such as the glycolytic ones that made up 30% of the proteins detected in the 2-D DIGE approach, and not those that are less abundant, such as those involved in e.g. transcription.

## Concluding Remarks

Comparative proteomics of free and encapsulated *S. cerevisiae* CBS8066 revealed numerous changes in the cells arising from the encapsulation. Most changes could be attributed to stricter anaerobic conditions and nutrient starvation of cells arising from mass transfer limitations into the interior of the cell pellet inside the capsule. Notable were for example the up-regulation of proteins related to trehalose and glycogen synthesis and utilization, alcohol dehydrogenases and many stress-related proteins, and the down-regulation of protein synthesis-related proteins. These changes mirrored the increased ethanol and decreased biomass yields compared to the free cells and also verified, on the protein level, previously reported findings of increased thermotolerance and increased levels of trehalose and glycogen inside similarly encapsulated cells. A number of changes in protein content that occurred due to the encapsulation can also be ascribed as contributing factors to the increased resistance of encapsulated cells towards furfural, one of the major lignocellulose-derived inhibitors.

## Supporting Information

Figure S1 **Heat map showing the proteomic differences between free and encapsulated** ***S. cerevisiae*** **.** Heat map of the 211 significantly changed proteins between three biological replicates each of encapsulated (E1–3) and free (F1–3) yeast, with ratios normalized to the average value of the free yeast and converted to log_2_-space for centring to zero. Clusters were computed using the default settings of heatmap.2 in the gplots package of R [47]. The column clustering shows the biological homogeneity among the replicates as well as the large differences between free and encapsulated yeast, while the row clustering shows similarities in protein abundance change among the regulated proteins. Missing value is shown in white.
(TIF)

Figure S2 **Proteome based pair-wise comparison of encapsulated and free** ***S. cerevisiae*** **.** Volcano plot illustrating the distribution of all proteins identified with the nLC-MS/MS approach with protein names shown for statistically regulated proteins. Significantly up- and down-regulated proteins (|fold change| ≥1.3, x-axis; FDR adjusted p value≤0.05, y-axis) are highlighted in green and red respectively. Statistically up- and down-regulated proteins with non-significant biological changes (|fold change| <1.3) are shown in light green and orange, respectively, and proteins with non-significant differences between the free and encapsulated yeast are shown in grey.
(TIF)

Figure S3 **GO functional enrichment analysis of differentially expressed proteins in free and encapsulated** ***S. cerevisiae*** **.** GO categories identified by TANGO, showing enriched categories and the percentage of the up- or down-regulated proteins belonging to the respective category above each bar.
(TIF)

Figure S4 **Promoter enrichment analysis of differentially expressed proteins in free and encapsulated** ***S. cerevisiae*** **.** A promoter enrichment analysis (p values are shown above the bars) of the genes coding for regulated proteins showed that the genes controlled by the transcription factor *ABF1*, for example controlling the expression of many ribosomal proteins, were over-represented among proteins down-regulated (light grey) in the encapsulated yeast. Enriched promoter usage among the up-regulated proteins (dark grey) in the encapsulated yeast were instead seen among the stress sensitive promoters *MSN2* and *4*, as well as the stress response element, *STRE*, and *ADR1*, required for transcription of genes necessary for ethanol, glycerol and fatty acid utilization, mirroring the starvation response from lack of glucose in the inner of the capsule.
(TIF)

Methods S1 Protein sample preparation, nLC-MS/MS and 2-D DIGE methods.
(DOCX)

Table S1 Proteins identified by the nLC-MS/MS approach.
(XLSX)

Table S2 Functional classification and cellular localization of proteins identified by the nLC-MS/MS approach.
(XLSX)

Table S3 Proteins identified by the 2-D DIGE approach.
(XLSX)
