# Supplementary figures and images for: Proteomic Analysis of the Increased Stress Tolerance of Saccharomyces cerevisiae Encapsulated in Liquid Core Alginate-Chitosan Capsules
Source: PLoS One. 2012 Nov 9;7(11):e49335. doi: 10.1371/journal.pone.0049335 (PMC3494678; doi:10.1371/journal.pone.0049335)

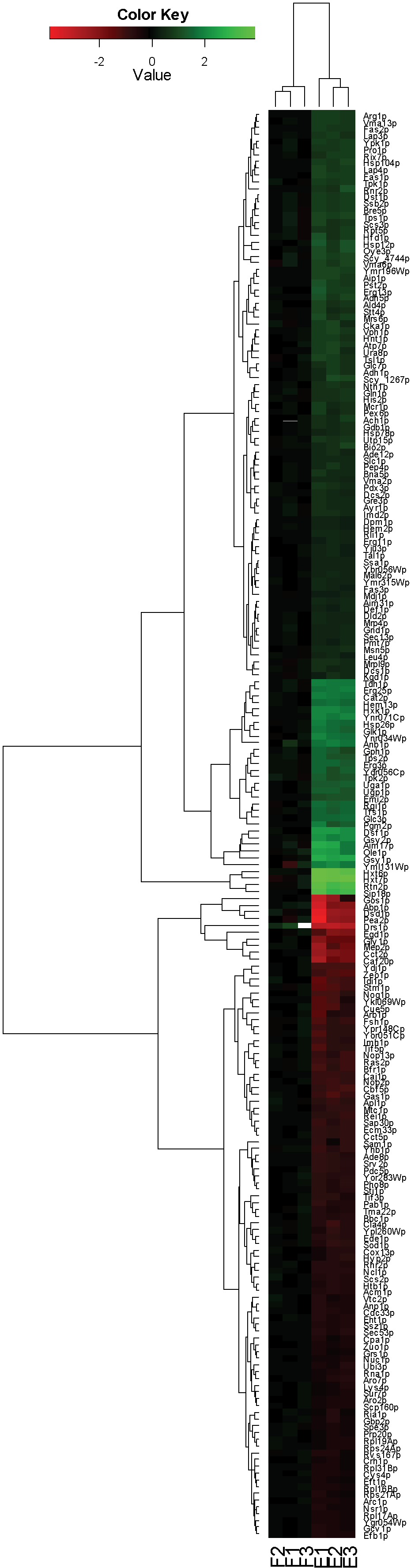

Supplement: Figure S1 — Heat map showing the proteomic differences between free and encapsulated S. cerevisiae . Heat map of the 211 significantly changed proteins between three biological replicates each of encapsulated (E1–3) and free (F1–3) yeast, with ratios normalized to the average value of the free yeast and converted to log2-space for centring to zero. Clusters were computed using the default settings of heatmap.2 in the gplots package of R [47]. The column clustering shows the biological homogeneity among the replicates as well as the large differences between free and encapsulated yeast, while the row clustering shows similarities in protein abundance change among the regulated proteins. Missing value is shown in white. (TIF) [file pone.0049335.s001.tif]

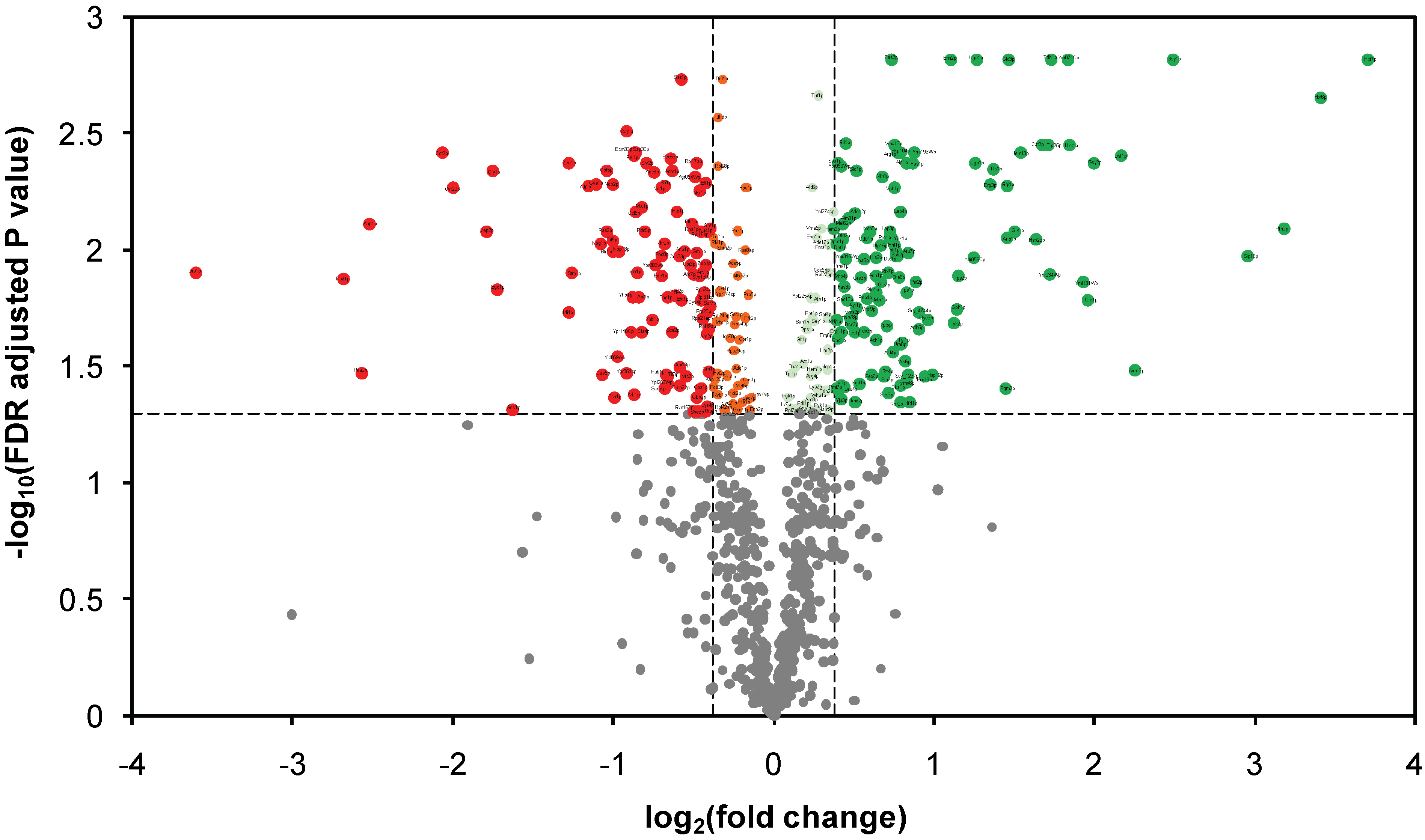

Supplement: Figure S2 — Proteome based pair-wise comparison of encapsulated and free S. cerevisiae . Volcano plot illustrating the distribution of all proteins identified with the nLC-MS/MS approach with protein names shown for statistically regulated proteins. Significantly up- and down-regulated proteins (|fold change| ≥1.3, x-axis; FDR adjusted p value≤0.05, y-axis) are highlighted in green and red respectively. Statistically up- and down-regulated proteins with non-significant biological changes (|fold change| <1.3) are shown in light green and orange, respectively, and proteins with non-significant differences between the free and encapsulated yeast are shown in grey. (TIF) [file pone.0049335.s002.tif]

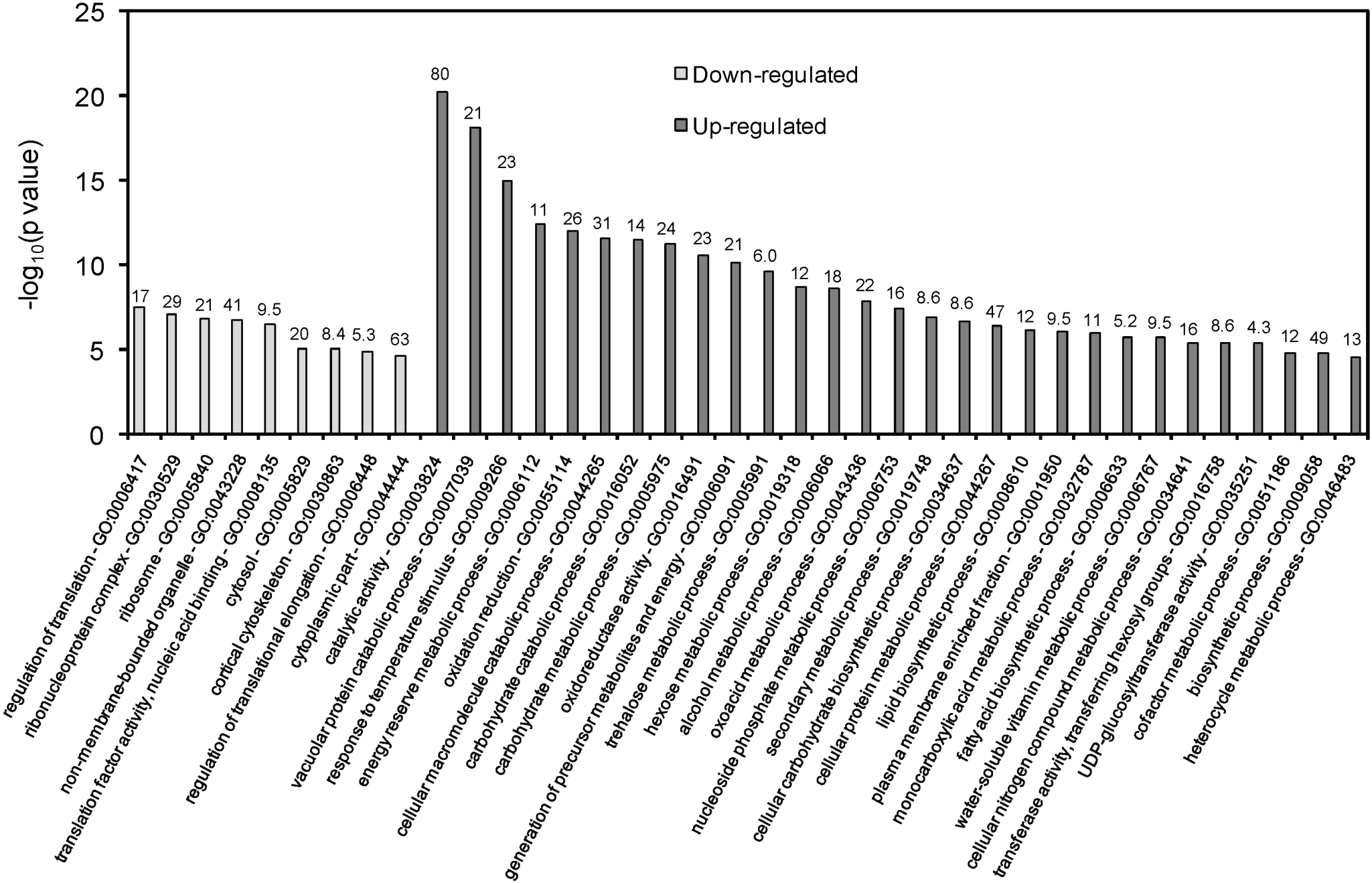

Supplement: Figure S3 — GO functional enrichment analysis of differentially expressed proteins in free and encapsulated S. cerevisiae . GO categories identified by TANGO, showing enriched categories and the percentage of the up- or down-regulated proteins belonging to the respective category above each bar. (TIF) [file pone.0049335.s003.tif]

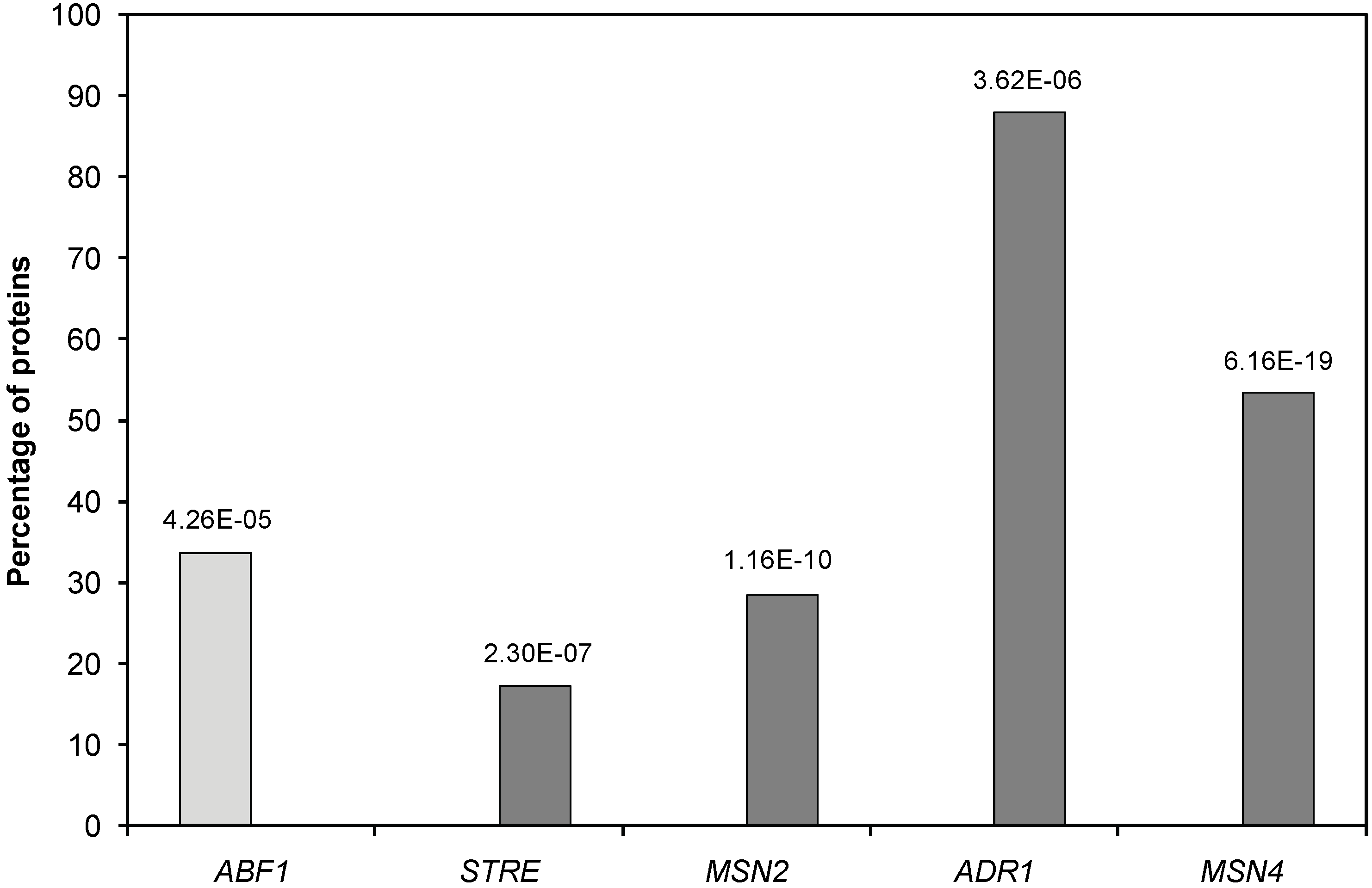

Supplement: Figure S4 — Promoter enrichment analysis of differentially expressed proteins in free and encapsulated S. cerevisiae . A promoter enrichment analysis (p values are shown above the bars) of the genes coding for regulated proteins showed that the genes controlled by the transcription factor ABF1, for example controlling the expression of many ribosomal proteins, were over-represented among proteins down-regulated (light grey) in the encapsulated yeast. Enriched promoter usage among the up-regulated proteins (dark grey) in the encapsulated yeast were instead seen among the stress sensitive promoters MSN2 and 4, as well as the stress response element, STRE, and ADR1, required for transcription of genes necessary for ethanol, glycerol and fatty acid utilization, mirroring the starvation response from lack of glucose in the inner of the capsule. (TIF) [file pone.0049335.s004.tif]
