# Supplementary material for: Proteomic Analysis of the Increased Stress Tolerance of Saccharomyces cerevisiae Encapsulated in Liquid Core Alginate-Chitosan Capsules
Source: PLoS One. 2012 Nov 9;7(11):e49335. doi: 10.1371/journal.pone.0049335 (PMC3494678; doi:10.1371/journal.pone.0049335)
Supplement: Methods S1 — Protein sample preparation, nLC-MS/MS and 2-D DIGE methods. (DOCX) [file pone.0049335.s005.docx]

**Methods S1**

# Supplementary information to:

# Proteomic analysis of the increased stress tolerance of *Saccharomyces cerevisiae* encapsulated in liquid core alginate-chitosan capsules

Johan O. Westman^1,2^, Mohammad J. Taherzadeh^1^, Carl Johan Franzén^2,§^

^1^ School of Engineering, University of Borås, 501 90 Borås, Sweden

^2^ Industrial biotechnology, Chalmers University of Technology, 412 96 Göteborg, Sweden

^§^Corresponding author

**Protein sample preparation**

Yeast cell protein extracts were prepared as follows. Yeast cell pellets were resuspended and washed once in 1 ml incubation buffer (IB) (20% sucrose, 50 mM 3-(N-morpholino)propanesulfonic acid (MOPS) and 10 mM MgCl_2_ at pH 7.0). After centrifugation the cells were resuspended in 400 µl IB and Nuclease Mix (GE Healthcare), 1:100 was added. The yeast cells were disrupted using a FastPrep (MP Biomedicals) and lysing matrix C (glass beads) at 5 x 40 s, 6.0 m s^-1^. The homogenate was incubated at 37°C for 15 min to digest nucleic acids and then centrifuged at 15,000 g for 10 min. The supernatant was transferred to tube #2. The pellet was treated with 200 µl lysis buffer (LB), (7 M urea, 2 M thiourea, 50 mM Tris/HCl, 50 mM 3-[(3-Cholamidopropyl)dimethylammonio]-1-
propanesulfonate (CHAPS) and 50 mM sodium dodecyl sulphate at pH 8.0), on a shaker (1500 rpm) for 30 min. After centrifugation the supernatant was transferred to tube #2. This step was repeated once with the addition of 100 mM dithiothreitol. The pooled supernatants were at this stage divided for 2-D DIGE and quantitative nLC-MS/MS. Protein concentrations were determined using Pierce 660 nm Protein Assay (Thermo). All samples were cleaned up using the ProteoExtract Protein Precipitation Kit prior to nLC-MS/MS or 2-D DIGE analysis.

**Sample preparation for nLC-MS/MS**

Precipitated protein pellets containing 100 µg of each sample (three biological replicates from free and three from encapsulated yeast) were dissolved in 20 µl lysis buffer containing 8 M urea, 4% 3-[(3-cholamidopropyl)dimethylammonio]-1-propanesulfonate (CHAPS), 0.1% sodium dodecyl sulphate (SDS) and 10 mM ethylenediaminetetraacetic acid (EDTA). Each sample was diluted four times with 40 µl 0.5 M tetraethylammonium bromide (TEAB) and 20 µl water followed by addition of 1 µl 2% SDS and reduction of the samples using Tris(2-carboxyethyl)phosphine (TCEP). The samples were alkylated using methyl methanethiosulfonate (MMTS) before digestion with 10 µl trypsin, 0.4 ug ul^-1^ (Promega). Peptides in each sample were labelled with the TMT^®^ reagent 126, 127, 128, 129, 130 and 131 respectively, following the manufacturer’s instructions (Thermo Fisher Scientific). The samples were dried in a SpeedVac, dissolved in mobile phase (25 mM ammonium formate in 25% acetonitrile (ACN)) and pooled in one tube. Fractionation by strong cation exchange chromatography of the TMT^®^-labelled peptides was performed as described previously [[1](#_ENREF_1)], with slight changes in the gradient; 0-20% B (500 mM ammonium formate, pH 2.8, 20% ACN) for 20 min; 20-100% B for 20 min, yielding 20 peptide-containing fractions.

**LC-MS/MS analysis, database search and quantitation**

The twenty peptide-containing fractions were desalted on PepClean C18 spin columns according to the manufacturer’s instructions (Thermo Fisher Scientific, Inc., Waltham, MA, USA). The fractions were resuspended in 0.1% formic acid and analysed on a LTQ-Orbitrap Velos (Thermo Fisher Scientific, Inc., Waltham, MA, USA) ) interfaced with an in-house constructed nano-LC column. Two-micro liter sample injections were made with an Easy-nLC autosampler (Thermo Fisher Scientific, Inc., Waltham, MA, USA), running at 200 nL min^-1^. The peptides were trapped on a precolumn (45 x 0.075 mm i.d.) and separated on a reversed phase column, 200 x 0.075 mm, packed in-house with 3 μm Reprosil-Pur C18-AQ particles. The gradient was as follows; 0-60 min 5-25% ACN, 0.1% formic acid, 60-70 min 25-80% ACN, 0.1% formic acid and the last 10 min at 80% ACN, 0.1% formic acid. LTQ-Orbitrap Velos settings were: spray voltage 1.4 kV, 1 microscan for MS1 scans at 60000 resolution (m/z 400), full MS mass range m/z 400-2000. The LTQ-Orbitrap Velos was operated in a data-dependent mode, that is, one MS1 FTMS scan precursor ions followed by HCD (high energy collision dissociation), MS2 scans of the ten most abundant doubly or triply protonated ions in each FTMS scan. The settings for the MS2 were as follows: 1 microscans for HCD-MS2 at 7500 resolution (at m/z 400), mass range m/z 100-2000 with a collision energy of 45%.

MS raw data files from the 20 fractions for the 6-plex TMT^®^ set were merged for identification and relative quantification using Proteome Discoverer version 1.2 (Thermo Fisher Scientific, Inc., Waltham, MA, USA). Database search was performed by Mascot search engine using the following criteria: *S. cerevisiae* in Swiss-Prot version October 2010; MS peptide tolerance 5 ppm; MS/MS tolerance 0.05 Da; trypsin digestion allowing 2 missed cleavages with variable modifications methionine oxidation and cysteine carbamidomethylation, and fixed modifications N-terminal TMT6plex and lysine TMT6plex. The protein identification threshold was set to 99% confidence using the FDR 1% method via decoy data base search [[2](#_ENREF_2),[3](#_ENREF_3)] and identified proteins were grouped by sharing the same sequences to minimize redundancy. Protein isoforms or homologues were grouped if they shared the detected peptide sequence and the unique peptide/s for a specific isoform was not detected.

  For tandem mass tag quantification, the ratios of the TMT^®^ reporter ion intensities in MS/MS spectra (m/z 126-131) from raw data sets were used to calculate fold changes (FC) between samples. Ratios were derived by Proteome Discoverer version 1.2 using the following criteria: fragment ion tolerance was 50 ppm for the most confident centroid peak; TMT^®^ reagent purity correction factors were used; and missing values were replaced with minimum intensity. Only peptides unique for a given protein or protein group were considered for relative quantitation, excluding those common to other iso-forms or proteins of the same family. The protein ratio in the individual encapsulated yeast sample was derived from the average of the relative quantification in the free cell samples. Protein ratios were calculated as the median of all peptides belonging to that protein/protein group per sample. Experimental bias (the difference in the total observed protein abundance between two or more samples) was corrected by normalisation to the protein median in each sample. Assuming that in real samples most of the proteins are not regulated, the intensity of the median protein in one sample should be the same as the intensity of the median protein in another. The nLC-MS/MS data associated with this manuscript may be downloaded from ProteomeCommons.org Tranche using the following hash:

t7gxXL8oO/kgqNEGa2zWDwUVQKnEa90+l/W++pR6JKuwNXrY1m7hHCrqnVsNn6M0pv3w0aebnXgnl8vXv6N+YlyNFHcAAAAAAAACug==.

The results were exported into Excel for manual data interpretation.

**Data analysis, nLC-MS/MS**

The fold change (FC) of the expression of each protein was obtained by dividing the average protein ratio of each protein in the encapsulated yeast with the average protein ratio in the free yeast sample. Fold changes less than one were inverted and given a negative sign. A t-test was used for pair-wise comparison with Benjamini-Hochberg’s method to adjust for multiple testing. A false discovery rate cut-off value of the adjusted p value ≤ 0.05 was chosen for statistical significance. The criterion for biologically significant changes was set to |FC| ≥ 1.3. Information on proteins was referenced from the *Saccharomyces* Genome Database (http://www.yeastgenome.org/), as well as from KEGG (<http://www.genome.jp/kegg/pathway.html>) for metabolic pathway reconstructions. Functional categorization and subcellular localization of proteins were carried out with FunCatDB Software (<http://mips.helmholtz-muenchen.de/proj/funcatDB/>). A heat map was created in R [[4](#_ENREF_4)] using heatmap.2 in the gplots package. Functional enrichment analysis and promoter analysis of regulated proteins were done by TANGO (Tool for Analysis of GO enrichments) and PRIMA (Promoter Integration in Microarray Analysis) respectively, that are incorporated in EXPANDER 5.2 (EXPression ANalyzer and DisplayER) [[5](#_ENREF_5)].

**Fluorescent dye labelling, 2-DE and image analysis**

The comparison between encapsulated cells and free cells was also performed by 2-D DIGE analyses [[6](#_ENREF_6)] across 5 gels, using the same pooled-sample internal standard, equimolecular mixture of all the samples, in all gels. The samples were G-Dye labelled according to the manufacturers standard protocol (NH DyeAGNOSTICS), in 7 M Urea, 2 M thiourea, 4% CHAPS, 30 mM Tris/HCl, pH 8 using 400 pmol of dye reagent for 50 µg of sample protein. Individual samples were labelled with G-Dye200 or G-Dye300 dyes using dye switching, while the internal standard was always G-Dye100 labelled.

Isoelectric focusing was done in 24 cm pH 3-10 Nonlinear Imobiline DryStrips (GE Healthcare) on an Ettan IPGphore. The second dimension was run on an Ettan DALT II in in-house made 1 mm polyacrylamide (T = 11%, C = 2.6%) Bis-Tris gels with standard MOPS cathode buffer and acetic acid/diethanol amine anode buffer. After 2D electrophoresis, the gels were scanned using the VersaDoc MP 4000 (BioRad) using excitation/emission wavelengths specific for the different G-Dyes (498 and 524, 554 and 575, and 648 and 663 nm for G-Dye100, G-Dye200 and G-Dye300 respectively). Gel images were analysed using the Progenesis SameSpots software ver. 4.1 (Nonlinear Dynamics) for spot detection, spot quantification, inter-gel matching, and statistics. Spots with significant differences (Anova p < 0.05) were selected for further identification.

**Spot picking and in-gel protein digestion**

A preparative gel of pooled samples to a total protein concentration of 450 µg stained with RuBPS (Ruthenium (II) tris (bathophenantroline disulfonate), RubiLAB, Switzerland) was used for protein identification. Selected protein spots, were picked, transferred to a micro-titre plate and trypsinated in the Ettan Spot-handling Workstation (GE Healthcare). The method for in-gel protein digestion with trypsin described by Shevchenko et al. [[7](#_ENREF_7)] was applied with some minor modifications. Briefly, the gel pieces were destained by washing three times in 25 mM NH_4_HCO_3_ in 50% CH_3_OH and once in 70% CH_3_CN. Gel pieces were dried and incubated with digestion buffer (50 mM NH_4_HCO_3_, 10 ng µl^-1^ trypsin (Promega)) at 37°C for 4 h. Peptides were extracted in 50% CH_3_CN / 0.5% trifluoroacetic acid, transferred to a new plate, dried and dissolved in 30% CH_3_CN / 0.1% TFA. The peptides were spotted onto an AnchorChip (Bruker) target using α-cyano-4-hydroxy-cinnamic acid as matrix.

**MALDI-TOF MS/MS.**

Selected samples were analysed on a MALDI TOF/TOF (Ultraflextreme, Bruker Daltonics, Bremen, Germany) instrument operated in the positive ion mode. The analysed mass range was 700 - 4,000 Da with ion suppression up to 600 Da. MS and MS/MS analyses were performed automatically. For MS analysis, 2,000 single-shot spectra were accumulated by recording 200-shot spectra at 10 random positions using fixed laser attenuation. Selection of precursor ions for MS/MS was performed using the Warp-LC software (Bruker Daltonics). For MS/MS analysis 2,000 single shots spectra were recorded for the precursors and 4,000 for the fragment ion spectra. Peptide and tandem mass spectra were searched by MASCOT against *S. cerevisiae* in the Swiss-Prot database. The search parameters were set to: MS accuracy 50 ppm, MS/MS accuracy 0.7 Da, one missed cleavage by trypsin allowed, fixed propionamide modification of cysteine and variable modification of oxidized methionine. Significant protein hits (for peptide mass fingerprinting p ≤ 0.05 and for MS/MS at least two peptides with p ≤ 0.05) were during the analysis separated regarding to whether they were the only significant hit in one spot or if there were more than one protein detected with significance in the spot (co-migrating) since this has implications on the ratios assigned to the respective protein.

**References**

1. O'Neil S, Sitkauskiene B, Babusyte A, Krisiukeniene A, Stravinskaite-Bieksiene K, et al. (2011) Network analysis of quantitative proteomics on asthmatic bronchi: effects of inhaled glucocorticoid treatment. Resp Res 12: 124.

2. Elias JE, Gygi SP (2007) Target-decoy search strategy for increased confidence in large-scale protein identifications by mass spectrometry. Nat Methods 4: 207-214.

3. Huttlin EL, Hegeman AD, Harms AC, Sussman MR (2007) Prediction of error associated with false-positive rate determination for peptide identification in large-scale proteomics experiments using a combined reverse and forward peptide sequence database strategy. J Proteome Res 6: 392-398.

4. R Development Core Team (2012) R: A language and environment for statistical computing. R Foundation for Statistical Computing, Vienna, Austria. ISBN 3-900051-07-0, URL <http://www.R-project.org/>

5. Shamir R, Maron-Katz A, Tanay A, Linhart C, Steinfeld I, et al. (2005) EXPANDER - an integrative program suite for microarray data analysis. BMC Bioinformatics 6: 232.

6. Alban A, David SO, Bjorkesten L, Andersson C, Sloge E, et al. (2003) A novel experimental design for comparative two-dimensional gel analysis: Two-dimensional difference gel electrophoresis incorporating a pooled internal standard. Proteomics 3: 36-44.

7. Shevchenko A, Wilm M, Vorm O, Mann M (1996) Mass spectrometric sequencing of proteins from silver-stained polyacrylamide gels. Anal Chem 68: 850-858.
